# EXAMINING LINKAGES BETWEEN NEIGHBORHOOD DISADVANTAGE AND SUBCLINICAL CARDIOVASCULAR DISEASE

**DOI:** 10.1093/geroni/igac059.453

**Published:** 2022-12-20

**Authors:** Regina Wright, Alexa Allan, Alyssa Gamaldo, Adrienne Aiken Morgan, Anna Lee

**Affiliations:** University of Delaware, Newark, Delaware, United States; The Pennsylvania State University, University Park, Pennsylvania, United States; Pennsylvania State University, State College, Pennsylvania, United States; University of North Carolina at Chapel Hill, Chapel Hill, North Carolina, United States; North Carolina A&T State University, Greensboro, North Carolina, United States

## Abstract

Limited research has examined the extent neighborhood disadvantage relates to subclinical CVD such as carotid atherosclerosis, arterial stiffness, and endothelial dysfunction which are key, understudied CVD risk markers. Our objective was to examine associations between neighborhood disadvantage and subclinical CVD and whether associations vary by age, sex, race, and education. The analysis included 165 Black and White older adults (mean age=64.5y). Neighborhood disadvantage was characterized using the Area Deprivation Index (ADI). Multivariable regression analysis, adjusted for age, sex, race, education, and depression, showed that national and state ADI were significant, inverse predictors of carotid atherosclerosis (near wall IMT; p=.02) and state ADI was a significant, inverse predictor of flow-mediated dilation (p=.04). A significant interaction between state and national ADI and education predicting carotid IMT emerged, but the simple slopes were non-significant (p=.02, .03). Future research should consider the role of neighborhood as a predictor of early cardiovascular risk.

